# Analysis of Sex and Gender Reporting Policies in Preeminent Biomedical Journals

**DOI:** 10.1001/jamanetworkopen.2022.30277

**Published:** 2022-08-31

**Authors:** Lorin A. Bibb, Brian D. Adkins, Garrett S. Booth, K. Maureen Shelton, Jeremy W. Jacobs

**Affiliations:** 1 Department of Dermatology, University of Connecticut Health, Farmington; 2 Division of Transfusion Medicine and Hemostasis, Department of Pathology, University of Texas Southwestern, Dallas; 3 Department of Pathology, Microbiology and Immunology, Vanderbilt University Medical Center, Nashville, Tennessee; 4 Department of Psychiatry and Psychology, Mayo Clinic, Rochester, Minnesota; 5 Department of Laboratory Medicine, Yale School of Medicine, New Haven, Connecticut

## Abstract

This cross-sectional study evaluates prominent journals’ sex and gender reporting guidelines.

## Introduction

The National Academies of Sciences, Engineering, and Medicine recently emphasized the importance of collecting sex and gender data in research, and avoiding conflation of these variables.^1^ Sex is defined as a biological attribute encompassing chromosomes and reproductive anatomy; gender refers to sociocultural roles and behaviors associated with one’s perception of themselves, including psychological, emotional, and behavioral identity.^1,2^ For accurate, equitable, and inclusive biomedical research, researchers must understand and distinguish between sex and gender to ensure the intended demographic is studied and reported, as these intricately linked variables uniquely influence health and disease.^3,4^ We evaluated prominent journals’ sex and gender reporting guidelines.

## Methods

Ethics committee approval was not required as all data are publicly available. Cross-sectional analysis adhering to the STROBE guidelines of the 20 journals with the highest 2020 impact factor (IF)^5^ for each of the 10 largest US medical specialties^6^ (eTable 1 in the Supplement) was performed to determine if journals: (1) have a sex and/or gender reporting policy; (2) distinguish between or define sex and gender; (3) require researchers to report their methods for determining sex and/or gender; and (4) require collection of both sex and gender.

All-time journal citations, journal founding date, and perceived gender (via online pronouns [he/she/they] or photographs if unavailable) of journal editor-in-chief (EIC) (April 1, 2022) were collected. Two-sided *P* < .05 was considered statistically significant. Statistical analysis was performed using GraphPad PRISM version 9.2.0 (GraphPad Software). Additional methodological details including statistical analysis are available in eTable 2 in the Supplement.

## Results

Author guidelines for 190 journals were analyzed (Table 1).^6^ Ten journals are among the top 20 for 2 different specialties. Among these 190 journals, 65 (34%) state a policy for reporting sex and/or gender in their author guidelines; 46 (24%) explicitly distinguish between or define the terms *gender* and *sex*; 31 (16%) recommend or require researchers to report their methods for determining sex and gender; and 3 (2%) require researchers to report both sex and gender demographics.

**Table 1.** Characteristics of Analyzed Medical Specialties and Associated Journals

Specialty	Total active physicians 2019^a^	Total EIC positions	Total women EIC, No. (%)	Mean (SD)	
				2020 IF^b^	Journal age, y
General medicine/internal medicine	120 171	21	8 (38.1)	24.8 (25.6)	86.2 (72.0)
Pediatrics	60 618	22	5 (22.7)	5.7 (3.2)	48.8 (30.7)
Emergency medicine	45 202	22	1 (4.5)	3.2 (1.2)	30.5 (13.4)
Obstetrics and gynecology	42 720	22	4 (18.2)	5.7 (2.8)	51.9 (35.7)
Anesthesiology	42 267	21	0 (0)	5.0 (2.1)	49.2 (29.8)
Psychiatry	38 792	21	3 (14.3)	13.4 (10.2)	53.3 (47.5)
Radiology	28 025	27	5 (18.5)	7.9 (2.3)	35.9 (20.3)
Surgery	25 564	21	3 (14.3)	7.7 (2.6)	58.8 (43.9)
Cardiology	22 521	20	2 (10)	14.9 (8.0)	32.8 (24.4)
Ophthalmology	19 312	23	2 (8.7)	5.7 (4.2)	61.4 (49.8)
Total^c^	445 192	209	31 (14.8)	9.5 (11.3)	51.6 (42.8)

Abbreviations: EIC, editor-in-chief; IF, impact factor.

^a^ Data from the Association of American Medical Colleges.^6^

^b^ Data from Journal Citation Reports.^5^

^c^ 190 unique journals.

Among the 10 specialties, obstetrics and gynecology had the largest percentage of top journals with a sex and gender reporting policy (65% [13 of 20]), while ophthalmology had the smallest (25% [5 of 20]). One specialty (general medicine/internal medicine: 45%) had greater than 20% of journals that instructed researchers to report their methods for determining sex and gender in studies.

There was no significant difference in mean IF, mean journal age, or EIC perceived gender between journals that have a sex and/or gender reporting policy and those that do not (Table 2). Similarly, there was no difference in journal IF, mean journal age, or EIC perceived gender and the presence of sex and gender definitions in author guidelines. Conversely, journals that require reporting of methods used to determine sex and/or gender have a significantly higher IF (15.7 [95% CI, 9.5-21.9] vs 8.3 [95% CI, 6.9-9.7]) and a significantly greater proportion of EIC positions held by women (29.4% [95% CI, 16.7%-46.3%] vs 12.0% [95% CI, 7.9%-17.7%]; *P* = .02) than those that do not require methods reporting.

**Table 2.** Association Between Sex and Gender Reporting Policies and Journal Characteristics

Criteria	Stated sex and/or gender reporting policy			Distinguish between or define sex and gender			Require reporting of methods used to determine sex and/or gender			Require collection of both sex and gender		
	Yes^a^	No	Mean difference (95% CI)	Yes^a^	No	Mean difference (95% CI)	Yes^a^	No	Mean difference (95% CI)	Yes^a^	No	Mean difference (95% CI)
2020 IF,^b^ mean (SD)	10.8 (13.1)	8.8 (10.3)	2.0 (−1.4 to 5.5)	12.1 (15.4)	8.7 (9.6)	3.4 (−0.4 to 7.2)	15.7 (17.7)	8.3 (9.2)	7.4 (3.1 to 11.7)	15.7 (12.4)	9.4 (11.3)	6.3 (−6.7 to 19.3)
Journal age, mean (SD), y	50.5 (46.1)	52.2 (41.2)	1.7 (−11.3 to 14.7)	46.6 (46.8)	53.3 (41.5)	6.7 (−7.6 to 21.0	47.6 (51.8)	52.4 (41.0)	4.8 (−11.8 to 21.4)	32.3 (8.4)	52.0 (43.1)	19.7 (−29.5 to 68.9)
Women EIC, No./No. (%)	15/70 (21.4)	16/139 (11.5)	*P* = .07	11/50 (22)	20/159 (12.6)	*P* = .11	10/34 (29.4)	21/175 (12)	*P* = .02	1/3 (33.3)	30/206 (14.6)	*P* = .38

Abbreviations: EIC, editor-in-chief; IF, impact factor.

^a^ Includes journals that have an external link to a policy.

^b^ Data from Journal Citation Reports.^5^

## Discussion

There is a paucity of policies outlining appropriate collection and reporting of sex and gender variables, even among the most influential biomedical journals. Despite guidance from organizations that explicitly defines and differentiates these demographics,^2^ few journals distinguish between them, and even fewer recommend or require authors to report their methods for determining sex and gender.

This study’s limitations included the inability to account for the entirety of the gender spectrum. Although researchers must be held accountable for appropriate study design and reporting of sex and gender variables, one cannot assume unequivocal adherence to expectations when journals themselves do not have best practice guidelines for study design and reporting. Without these, future research risks inaccurate results, reduced applicability, and exclusion of groups of historically marginalized individuals from research.

## Notes

Supplement. **eTable 1.** Journals Analyzed in the Included Research Study **eTable 2.** Methodological Details for Variable Analysis of Journals
