# Supplementary material for: Analysis of Sex and Gender Reporting Policies in Preeminent Biomedical Journals
Source: JAMA Netw Open. 2022 Aug 31;5(8):e2230277. doi: 10.1001/jamanetworkopen.2022.30277 (PMC9434354; doi:10.1001/jamanetworkopen.2022.30277)
Supplement: Supplement. — eTable 1. Journals Analyzed in the Included Research Study eTable 2. Methodological Details for Variable Analysis of Journals [file jamanetwopen-e2230277-s001.pdf]

Supplemental Online Content

Bibb LA, Adkins BD, Booth GS, Shelton KM, Jacobs JW. Analysis of sex and gender reporting policies in preeminent biomedical journals. *JAMA Netw Open.* 2022;5(8):e2230277. doi:10.1001/jamanetworkopen.2022.30277

eTable 1. Journals Analyzed in the Included Research Study

eTable 2. Methodological Details for Variable Analysis of Journals

This supplemental material has been provided by the authors to give readers additional information about their work.

© 2022 Bibb LA et al. *JAMA Network Open.*

eTable 1. List of all journals analyzed by specialty

Specialty	Journals
General medicine/internal medicine	New England Journal of Medicine; Lancet; JAMA-Journal of the American Medical Association; Nature Reviews Disease Primers; BMJ-British Medical Journal; Annals of Internal Medicine; Lancet Digital Health; JAMA Internal Medicine; Journal of Cachexia Sarcopenia and Muscle; PLOS Medicine; Cochrane Database of Systematic Reviews; Journal of Internal Medicine; BMC Medicine; Journal of Travel Medicine; JAMA Network Open; Canadian Medical Association Journal; Medical Journal of Australia; Mayo Clinic Proceedings; Amyloid-Journal of Protein Folding Disorders; Translational Research
Pediatrics	JAMA Pediatrics; Lancet Child & Adolescent Health; Journal of the American Academy of Child and Adolescent Psychiatry; Pediatrics; Pediatric Allergy and Immunology; Archives of Disease in Childhood-Fetal and Neonatal Edition; Developmental Medicine and Child Neurology; Pediatric Diabetes; Journal of Adolescent Health; European Child & Adolescent Psychiatry; Journal of Pediatrics; Neonatology; Pediatric Obesity; Paediatric and Perinatal Epidemiology; Seminars in Fetal and Neonatal Medicine; Archives of Disease in Childhood; Pediatric Research; Pediatric Nephrology; Birth-Issues in Perinatal Care; Pediatric Critical Care Medicine
Emergency medicine	Annals of Emergency Medicine; World Journal of Emergency Surgery; Resuscitation; Burns & Trauma; Emergencias; European Journal of Trauma and Emergency Surgery; Academic Emergency Medicine; Prehospital Emergency Care; Scandinavian Journal of Trauma Resuscitation & Emergency Medicine; European Journal of Emergency Medicine; Emergency Medicine Journal; Injury-International Journal of the Care of the Injured; American Journal of Emergency Medicine; Western Journal of Emergency Medicine; Canadian Journal of Emergency Medicine; World Journal of Emergency Medicine; Emergency Medicine Clinics of North America; Emergency Medicine Australasia; BMC Emergency Medicine; Prehospital and Disaster Medicine
Obstetrics and gynecology	Human Reproduction Update; American Journal of Obstetrics and Gynecology; Obstetrics and Gynecology; Fertility and Sterility; Ultrasound in Obstetrics & Gynecology; Human Reproduction; BJOG-An International Journal of Obstetrics and Gynecology; Gynecologic Oncology; Best Practice & Research Clinical Obstetrics & Gynaecology; Journal of Gynecologic Oncology; Breast; Maturitas; Breast Cancer; Journal of Minimally Invasive Gynecology; Molecular Human Reproduction; Paediatric and Perinatal Epidemiology; Reproductive Biomedicine Online; Birth-Issues in Perinatal Care; Acta Obstetricia et Gynecologica Scandivavica; International Journal of Gynecology & Obstetrics
Anesthesiology	Journal of Clinical Anesthesia; British Journal of Anesthesia; Anesthesiology; Pain; Anaesthesia; Regional Anesthesia and Pain Medicine; Anesthesia and Analgesia; Canadian Journal of Anesthesia-Journal Canadien d Aneshtesie; Pain Physician; European Journal of Anesthesiology; Anaeshtesia Critical Care & Pain Medicine; Journal of Neurosurgical Anesthesiology; European Journal of Pain; Pain Medicine; Perioperative Medicine; Clinical Journal of Pain; Pain Practice; Minerva Anestesiologica; Current Opinion in Anesthesiology; Journal of Cardiothoracic and Vascular Anesthesia
Psychiatry	World Psychiatry; Lancet Psychiatry; JAMA Psychiatry; American Journal of Psychiatry; Psychotherapy and Psychosomatics; Molecular Psychiatry; Biological Psychiatry; Journal of Neurology Neurosurgery and Psychiatry; British Journal of Psychiatry; Schizophrenia Bulletin; Journal of Child Psychology and Psychiatry; Journal of the American Academy of Child and Adolescent Psychiatry; Evidence-Based Mental Health; Neuropsychopharmacology; Psychological Medicine; Brain Behavior and Immunity; Clinical Psychological Science; Epidemiology and Psychiatric Sciences; Journal of Behavioral Addictions; Bipolar Disorders
Radiology	JACC-Cardiovascular Imaging; Radiology; Journal of Nuclear Medicine; IEEE Transactions on Medical Imaging; European Journal of Nuclear Medicine and Molecular Imaging; Medical Image Analysis; Photoacoustics; Clinical Nuclear Medicine; Circulation-Cardiovascular Imaging; Ultrasound in Obstetrics & Gynecology; International Journal of Radiation Oncology Biology Physics; European Heart Journal-Cardiovascular Imaging; Neuroimage; Ultraschall in der Medizin; Radiotherapy and Oncology; Investigative

© 2022 Bibb LA et al. *JAMA Network Open.*

Specialty	Journals
	Radiology; Journal of Nuclear Cardiology; Seminars in Radiation Oncology; Journal of the American College of Radiology; Journal of Cardiovascular Magnetic Resonance
Surgery	JAMA Surgery; Annals of Surgery; Journal of Neurology Neurosurgery and Psychiatry; Journal of Heart and Lung Transplantation; Endoscopy; American Journal of Transplantation; Digestive Endoscopy; Hepatobiliary Surgery and Nutrition; European Journal of Vascular and Endovascular Surgery; Journal of Hepato-Biliary-Pancreatic Sciences; British Journal of Surgery; American Journal of Surgical Pathology; JAMA Otolaryngology-Head & Neck Surgery; Journal of the American College of Surgeons; International Journal of Surgery; Liver Transplantation; World Journal of Emergency Surgery; Annals of Surgical Oncology; Journal of Bone and Joint Surgery-American Volume
Cardiology	Nature Reviews Cardiology; European Heart Journal; Circulation; Journal of the American College of Cardiology; Circulation Research; Basic Research in Cardiology; European Journal of Heart Failure; JACC-Cardiovascular Imaging; JAMA Cardiology; JACC-Heart Failure; JACC-Cardiovascular Interventions; Cardiovascular Research; Journal of Heart and Lung Transplantation; Cardiovascular Diabetology; Circulation-Heart Failure; JACC-Basic to Translational Science; Progress in Cardiovascular Diseases; European Journal of Preventive Cardiology; Circulation-Cardiovascular Imaging; European Heart Journal-Cardiovascular Imaging
Ophthalmology	Progress in Retinal and Eye Research; Ophthalmology; JAMA Ophthalmology; Annual Review of Vision Science; Survey of Ophthalmology; American Journal of Ophthalmology; Ocular Surface; Investigative Ophthalmology & Visual Science; British Journal of Ophthalmology; Retina-The Journal of Retinal and Vitreous Diseases; Clinical and Experimental Ophthalmology; Eye; Current Opinion in Ophthalmology; Acta Ophthalmologica; Journal of Refractive Surgery; Ophthalmology and Therapy; Experimental Eye Research; Journal of Cataract and Refractive Surgery; Translational Vision Science & Technology; Eye and Vision

© 2022 Bibb LA et al. *JAMA Network Open.*

eTable 2. Methodological details for variable analysis of journals

Variable	Methodology
Perceived gender of editor-in-chief	Two authors (JWJ and LAB) who were non-blinded to the study hypothesis independently assessed the 190 journals, extracted the editor-in-chief (EIC) from each journal's masthead and coded the EIC perceived gender. Gender was determined via online review of pronouns (e.g., he/she/they). If unavailable, the author's photograph and name were used in combination to assign the perceived gender. Author consensus was planned for indeterminate cases; however, this was not required as interrater reliability was 100%. There were no instances of the use of "they" and other non-binary pronouns were not identified; thus, perceived gender is reported as binary.
Journal founding date	The journal founding date was extracted independently by two authors (JWJ and LAB) from journal websites with 100% agreement between authors.
Journal impact factor and all-time journal citations	Journal impact factor and all-time journal citations were collected by two authors (JWJ and LAB) from Journal Citation Reports by Clarivate Analytics with 100% agreement between authors.
Statistical analysis	
Statistical analysis was performed using GraphPad PRISM version 9.2.0 (GraphPad Software, LLC San Diego, CA, USA). An alpha of 0.05 was utilized to construct two-sided confidence intervals of differences between the mean impact factor and mean age of journals with and without: 1) a sex and/or gender reporting policy; 2) sex and gender definitions 3) a requirement for researchers to report their methods for determining sex and/or gender; 4) a requirement for the collection of both sex and gender.<br><br>Fisher exact test was utilized to compare the proportion of women editors-in-chief of journals with and without: 1) a sex and/or gender reporting policy; 2) sex and gender definitions 3) a requirement for researchers to report their methods for determining sex and/or gender; 4) a requirement for the collection of both sex and gender.<br><br>The hypothesis tested was that journals with these policies in place would have a higher impact factor and/or be founded earlier.	

© 2022 Bibb LA et al. *JAMA Network Open.*
